# Gram-Negative Microbiota Derived from Trout Fished in Slovakian Water Sources and Their Relationship to Postbiotics

**DOI:** 10.3390/pathogens14070644

**Published:** 2025-06-28

**Authors:** Andrea Lauková, Anna Kandričáková, Jana Ščerbová, Monika Pogány Simonová, Rudolf Žitňan

**Affiliations:** 1 Centre of Biosciences of the Slovak Academy of Sciences, Institute of Animal Physiology, Šoltésovej 4-6, 040 01 Košice, Slovakia; kandricakova@saske.sk (A.K.); scerbova@saske.sk (J.Š.); simonova@saske.sk (M.P.S.); 2 Parasitological Institute, The Slovak Academy of Sciences, Hlinkova 3, 040 01 Košice, Slovakia; 3 National Agricultural and Food Centre, Research Institute for Animal Production, Hlohovecká 2, 951 41 Nitra-Lužianky, Slovakia; rudolf.zitnan@gmail.com

**Keywords:** trout, Gram-negative species, variability postbiotics, treatment

## Abstract

Regarding the trout microbiota, most information is focused on lactic acid bacteria, which can show beneficial properties. However, in trout farming, mostly pathogenic Gram-positive species were reported, such as *Staphylococcus aureus*, *Listeria monocytogenes*, and/or *Clostridium* spp. In this study, free-living trout were analyzed for Gram-negative microbiota that can cause loss as disease-stimulating agents. Bacteriocin postbiotics should be one of the approaches used to eliminate these agents. In total, 21 strains of different species isolated from the intestinal tract of 50 trout in Slovakia (*Salmo trutta* and *Salmo gairdnerii*) were taxonomically allotted into 13 species and 9 genera. This method showed variability in microbiota identified using MALDI-TOF mass spectrometry with the following species: *Acinetobacter calcoaceticus*, *Citrobacter gillenii*, *Citrobacter freundii*, *Escherichia coli*, *Hafnia alvei*, *Kluyvera cryocrescens*, *K. intermedia*, *Leclercia adecarboxylata*, *Raoultella ornithinolytica*, *Pseudomonas fragi*, *Ps. putida*, *Ps. lundensis*, *Ps. teatrolens*, and *Serratia fonticola*. Most strains were susceptible to the antibiotics used, reaching inhibitory zones up to 29 mm. On the other hand, 3 out of 21 strains (14%) were susceptible to nine enterocins- postbiotics (*Hafnia alvei* Hal281, *Pseudomonas putida* Pp391, and *Ps. fragi* Pf 284), with inhibitory activity in the range of 100–6400 AU/mL.

## 1. Introduction

Freshwater hosts a substantial source of animal protein in the human diet. Nowadays, the growing importance of aquaculture in the production of animal-originating proteins has been noted [1]. Trout can contribute to this source. It belongs to the numerous species of carnivorous freshwater ray-finned fishes. As is generally known, this fish species is taxonomically involved in the genus *Salmo* and the family Salmonidae. Trout as a species is classified as an oily fish and has been an important food fish for humans [2]. Oily fish refer to fish species with oil (fats) in soft tissues and in the coelomic cavity around the gut. Fillets of these fishes may contain up to 30% oil, although this figure varies both within and between species [2]. Oily fish can be contrasted with whitefish (e.g., cod), which contain oil only in the liver and in a much smaller overall quantity than oily fish. Oily fish meat is a good source of important fat-soluble vitamins such as vitamin A and D, and it is rich in omega-3 fatty acid (white fish also contain these nutrients but at a much lower concentration). For this reason, the consumption of oily fish rather than white fish can be more beneficial to humans, particularly in relation to heart diseases such as stroke and ischemic heart disease [2]. The omega-3 fatty acids in oily fish may help improve inflammatory conditions such as arthritis [2]. However, oily fish are known to carry higher levels of contaminants than white fish [2].

Nowadays, there is also increased concern about trout farming. The main challenges of small-scale farmers are seed availability and maintenance, growth, and reproduction functions. Fish use mostly dietary proteins continuously, and the availability of quality feeds is the main challenge faced by the farmers. However, there exist many factors from catch to processing that influence the natural condition of the fish when it is captured and handled [3]. The microbial aspect is among those factors.

The intestinal microbiota of fish (trout included) is often reported to be highly variable [4]. Of course, it depends on several factors, including environmental and temperature conditions. The majority of research is focused on lactic acid bacteria (LAB), which can show beneficial properties, including bacteriocinogenic characteristics [5]. The interest in the usage of beneficial LAB is associated mostly with fish farming in the form of trout production, which accounts for about 70% in Slovakia [5,6,7]. Regarding pathogens, mostly Gram-positive species have been mentioned, such as *Staphylococcus aureus*, *Listeria monocytogenes*, and/or *Clostridium* spp. [7]. Although there are existing microbial analyses of trout based on different identification systems and levels, there is still a gap in knowledge. However, in our case, free-living trout were analyzed on Gram-negative microbiota, because less interest is focused on mapping non-essential Gram-negative microbiota. Through farming, but also in free-living fish, massive losses can be caused by diseases due to pathogenic (mostly Gram-negative) bacteria [4,8,9]. In addition, the aquatic environment plays a preponderant role in the composition of the microbial community constituting the fish gut microbiota [1,9,10,11]. For example, Kim et al. [10] reported a high percentage abundance of the phyla Proteobacteria and Actinobacteria in rainbow trout. Three morphological types of Gram-negative, oxidase-positive bacteria were identified in fish by Grigoryan et al. [3].

Semwal et al. [9] also reported the representatives of Gammaproteobacteria as disease agents in aquaculture. Among them, the species *Aeromonas hydrophila* has been frequently detected [9], which was also referred to in our previous study [12]. This species is usually considered a secondary pathogen that infects fish that have already been infected with another pathogen, and/or as an opportunistic invader infecting fish under stress conditions. It is also an efficient biomarker of a stressed or polluted aquatic environment [9].

One strategy/approach on how to prevent/reduce contaminants in fish can be represented by probiotic/beneficial bacteria and/or substances produced by them—bacteriocins [5,13,14,15,16,17,18]. Nowadays, bacteriocins are part of the group of postbiotics. Postbiotics are defined as preparations of inanimate microbiota and/or their components, conferring health to the host [16]. They have been mostly explored as therapeutics in veterinary medicine for addressing bovine mastitis [19]. Another explored application of bacteriocins is as a preventive or growth promoter in broiler production for controlling pathogens or non-beneficial microbiota [19,20,21,22,23]. In this case, especially postbiotics, enterocins characterized in our laboratory were applied, e.g., enterocin (Ent) M, a thermo-stable small peptide produced by the environmental strain *E. faecium* AL41 (CCM8558), which was seen to achieve beneficial effects in rabbits [19,20,21,22,23]. Increased phagocytic activity in blood samples and a decrease in the fecal coliforms, *S. aureus*, and enterococci were noted after 21 days of the application of EntM in broiler rabbits. Moreover, higher daily weight gain was noted with EntM application, although no influence on feed conversion was seen [23].

Keeping in mind these benefits, and based on our previous in vitro and in vivo studies with postbiotics, one aim of this study was to show variability regarding the Gram-negative representatives derived from free-living trout originating from Slovakian water sources and to show if postbiotic (enterocin) treatment can inhibit them to support a sustainable health strategy in aquaculture, because some Gram-negative species can cause disease due to being a stimulating agent. Therefore, the use of postbiotics to inhibit them seems to be an alternative innovative approach following the One Health concept.

## 2. Materials and Methods

### 2.1. Sampling and Species Strain Identification

Fifty (*n* = 50) healthy trout (*Salmo trutta* and *Salmo gairdnerii*) were sampled from different sides of the regular pond Bukovec near Košice in eastern Slovakia and/or the river Čierny Váh in Central Slovakia in April of the years 2012–2015. Trout were supplied by individual fisherman who had obtained the appropriate permission for fishing to follow scientific research and exploratory purposes. Trout were fished based on the special fishing permit for scientific research and exploratory purposes (No. 42/2012, No. 21/2013, No. 4/2014, and No. 1/2015 provided by the Ministry of Environment of the Slovak Republic). The intestinal samples of trout were collected at the place of the trout fishing formerly described by Lauková et al. [12]. Samples were taken aseptically from the fish and placed into sterile Petri dishes, stored at 4 °C for approximately 4 h, and transported to the laboratory. They were treated using the standard microbial dilution method specified by the ISO (International Organization for Standardization). The samples were stirred in Ringer solution (1:9, pH 7.0, Merck, Darmstadt, Germany). The appropriate dilutions were plated onto different agars, such as CLED agar (Merck, Darmstadt, Germany), Mac Conkey agar for enterobacteriae (Difco, Sparks, MD, USA), and/or Cetrimide agar (Difco, USA). The agar plates were cultivated at 28–37 °C for 24–48 h according to the type of selected media. The grown colonies from the highest dilution were randomly picked up, their purity was controlled by plating on Trypticase soy agar with blood (Difco, USA), and their morphology was assessed. Strain identification was performed with the use of MALDI-TOF mass spectrometry (Matrix-Assisted Laser Desorption/Ionization Time of Flight Mass Spectrometry). This is an accurate method used in laboratory practices. This method has a very simple approach designed for protein detection and reliable technology for the precise identification of cultured microorganisms [12]. For MALDI-TOF mass spectrometry, a solitary colony of each isolate from Trypticase soy agar (supplemented with 3% defibrinated sheep blood, Difco, Sparks, MD, USA) was mixed with a matrix (α-cyano-hydroxycinnamic acid and trifluoroacetic acid). The suspension was spotted onto a MALDI plate and ionized with a nitrogen laser (wavelength of 337 mm; frequency of 20 Hz). This identification method is based on protein “fingerprints” (Bruker Daltonics, Biotyper 2.2. [12,24,25]), performed using a Microflex MALDI-TOF mass spectrometer, as previously described by Lauková et al. [12]. The results were evaluated according to the MALDI Biotyper 3.0 (Bruker Daltonics, Billerica, MA, USA) identification database. Taxonomic classification was assessed on the basis of highly probable species identification (a value score of 2.300–3.000), secure probable species identification/probable species identification (2.000–2.299), and on the basis of highly probable genus identification (1.700–1.999). Positive controls were those involved in the identification system. Identical colonies evaluated using the MALDI-TOF MS score value were excluded. Finally, 21 strains were used for the next testing. They were stored for analyses conducted with the Microbank system (Pro-Lab Diagnostic, Richmond, BC, Canada).

### 2.2. Antibiotic Phenotype of the Identified Species Strains

The commercial antibiotic discs supplied by Oxoid Ltd. (Basingstoke, UK) were used in the disc diffusion test. They were decided according to CLSI [26]. The following antibiotic discs were tested: clindamycin (2 µg), ampicillin (10 µg), penicillin G (10 IU), erythromycin, azithromycin (15 µg), chloramphenicol, tetracycline, amikacin (30 µg), azlocillin, mezlocillin, ticarcillin (75 µg), trimethoprim (85 µg), carbenicillin, piperacillin 100 µg), and gentamicin (120 µg). Broth cultures (in Trypticase soy broth, Difco) of tested strains (100 µL) were spread onto Mueller–Hinton agar (Bio-Rad, Bratislava, Slovakia) supplemented with 3% of sheep blood. The antibiotic discs were applied to the agar surface. The plates were incubated at 37 °C for 18 h. The inhibitory zones were evaluated and expressed in mm. They were reported according to the guidelines of the Clinical and Laboratory Standards Institute [15]. *E. coli* ATCC 25922 was the control strain.

### 2.3. Testing Gram-Negative Species Strains for Their Susceptibility to Postbiotics

The precipitates (partially purified substances) from our nine producing strains were used. Enterocin–dipeptide (Ent) A/P was produced by the environmental strain EK13 (CCM 7419 [27]). Ent A/P is a thermo-stable dipeptide with a molecular mass of 4830 Da. Its optimum production is associated with the pH range of 5.0–6.5 at 30 °C and 37 °C. The most active substance is produced in the logarithmic growth phase of the producer strain. It has a broad inhibitory spectrum. EntM, similarly to EntA/P, is produced by the environmental strain *E. faecium* AL41 = CCM8558. It is a thermo-stable, small peptide (molecular mass 4628 Da), with a broad antimicrobial spectrum [18]. Ent412, produced by the horse-derived strain *E. faecium* EF 412, indicates a broad inhibitory spectrum [28]. Ent4231, produced by the ruminal strain *E. faecium* CCM4231 [29], has been shown to have a broad antimicrobial spectrum [29]. It is susceptible to pronase and thermo-resistant. Ent55, produced by the poultry strain *E. faecium* EF [30] is a thermo-stable bacteriocin with the highest production in the late logarithmic growth phase, with optimal production at pH levels ranging from 7.0 to 9.0. Its inhibitory activity is mostly against Gram-positive microbiota. Ent2019 is produced by the rabbit-derived strain *E. faecium* 2019-CCM7420 [23], and Ent 9296 is from the silage strain *E. faecium* EF9296 [31]. EntEM41 is produced by the strain *E. faecium* EM41 from ostrich [22], and Durancin ED26E/7 is produced by the milk lump cheese-derived strain *E. durans* ED26E/7 [32,33]. Enterocins were prepared according to the previously reported protocols. They are class II enterocins. Testing was performed using the agar spot method according to De Vuyst et al. [34]. In brief, BHagar (Difco) was basic agar. Overnight, a broth culture of the EA5 strain (A_600_ up to 1.0) (200 µL) was applied into 0.7% BHagar, and the plate was overlaid. The appropriate dilutions of enterocins (in phosphate buffer, pH 6.5, 10 µL) were spotted on the agar plate surface. The plates were stored for 10 min in the fridge to diffuse the enterocin. Then, they were incubated at 37 °C overnight. The inhibitory activity was expressed in arbitrary units per mL (AU/mL). This means a two-fold dilution (in phosphate buffer pH 5.5) of the precipitate, which inhibited the growth of the indicator strain. The initial activity of the precipitate was measured against the principal indicator *E. avium* EA5—our strain (EntM, Ent2019, Ent 9296—25,600 AU/mL, EntA/P, Ent 55—51,200 AU/mL, Ent 412—204,800 AU/mL, ED26E/7 Ent 4231—6400 AU/mL, and EM41—800 AU/mL).

## 3. Results

### 3.1. Identified Species Strains

Broad species variability was found among trout-derived Gram-negative strains. A total of 21 strains were taxonomically allotted to 13 species and 9 genera. The following species were detected (Table 1): *Acinetobacter calcoaceticus*, *Citrobacter gillenii*, *Citrobacter freundii*, *Escherichia coli*, *Hafnia alvei*, *Kluyvera cryocrescens*, *K. intermedia*, *Leclercia adecarboxylata*, *Raoultella ornithinolytica*, *Pseudomonas fragi*, *Ps. putida*, *Ps. lundensis*, *Ps. teatrolens*, and *Serratia fonticola*. Six variable species strains (6) were identified, reaching a score value in the range of 2.300–3.000 (Table 1), meaning highly probable species identification. The species *Hafnia alvei* Hal401 (2.487), *Kluyvera intermedia* Ki382 (2.425), *K. intermedia* Ki 371 (2.326), *Raoultella ornithinolytica* Ro381 (2.468), *Pseudomonas putida* Pp391 (2.300), and *E. coli* EC361 (2.435) were also identified. Most of the strains were identified with a score in the range of 2.000–2.299 (Table 1), involving 11 different species identified based on secure probable species identification/probable species identification. A score of 1.700–1.999 was found for four strains, *Ps. fragii* Pf284 and Pf302, *Ps. teatrolens* Pt285, and *Leclercia adecarboxylata* LA381 (Table 1). The strains identified from trout sample No. 36 included four different species: *Acinetobacter calcoaceticus* AC362, *Escherichia coli* EC361, *Ps. lundensis* PL361, and *Serratia fonticola* SF361. The species *K. intermedia* Ki371, *Citrobacter gillenii* CG372, and *H. alvei* Hal 371 were detected in trout sample No. 37. Six species, such as *C. gillenii* CG382, *H. alvei* Hal382, *K. cryocrescens Kc383, K. intermedia* Ki382, *Leclercia adecarboxylata* LA381, and *R. ornithinolytica* Ro381, were derived from trout sample No. 38. The strains of the species *Ps. teatrolens* Pt285, *Ps. fragi* Pf284, and *H. alvei* Hal281 were also isolated from the same trout. In trout No. 40, *H. alvei* Hal401 and C. *freundii* CF402 were determined. In trout No. 30, *Ps. fragi* Pf302 was found. *H. alvei* Hal331 was detected in trout No. 33, and *Ps. putida* Pp391 was found in a sample of trout No. 39. The most prevalent Gram-negative species among trout were *Hafnia alvei* and different species of the genus *Pseudomonas* (Table 1). Trout No. 28 and 30 were caught in Čierny Váh; the others, No. 33–40 trout, were caught in the pond in Bukovec.

### 3.2. Susceptibility to Antibiotics and Postbiotics

The strains were treated with 15 antibiotics according to CLSI [26]. Twenty-one tested strains (21) were susceptible to mezlocillin, with an inhibitory zone size in the range of 15–23 mm. They were also susceptible to tetracycline (17–20 mm), chloramphenicol (22–27 mm), piperacillin (17–29 mm), gentamicin (16–26 mm), amikacin (10–22 mm), azlocillin (12–22 mm), and azithromycin (10–20 mm). In contrast, 21 strains were resistant to penicillin. Almost all strains (except Hal281) were resistant to erythromycin and clindamycin (Table 2). Altogether, Gram-negative bacteria tested were mostly susceptible to the antibiotic used; all were at least susceptible to 8 out of 15 antibiotics, and most were susceptible to 13 out of 15 antibiotics. Among the tested strains, three (3) were susceptible to 12 antibiotics (resistant to penicillin). Hal281 was the most susceptible (to 13 antibiotics out of 15 used). Nine strains were susceptible to 10 antibiotics, and seven strains were susceptible to 9 antibiotics. Even LA381 (Table 2) was susceptible to 8 antibiotics out of 15 tested. Only two strains were resistant to ticarcillin (LA381, Hal331).

Regarding postbiotic treatment, 3 out of 21 strains were susceptible to enterocins-postbiotics. The strains included *Hafnia alvei* Hal281, *Pseudomonas putida* Pp391, and *Ps. fragii* Pf 284 (Table 3). Pseudomonads were inhibited, with Ents reaching inhibitory activity of 100 AU/mL. However, *Hafnia alvei* Hal281 was the most susceptible. In the case of EntA/P, Ent9296, Ent412, Ent2019, and Durancin-like substance ED26E/7, inhibitory activity reached 6400 AU/mL. The other Ents inhibited Hal281, with inhibitory activity of 3200 AU/mL. This means that only 3 out of 21 different strain species (14%) were susceptible to nine of the postbiotics used. Eighty-six (86%) Gram-negative species strains were not inhibited by the Ents (postbiotics) used.

## 4. Discussion

The strain *Acinetobacter calcoaceticus* AC362 is part of the phylum Pseudomonata, class Gammaproteobacteria, order Pseudomonales, family Moraxellacae, and genus *Acinetobacter* [35]. To the same phylum and class, the species strain *Kluyvera cryocrescens* Kc383 and the strains *K. intermedia*, Ki382, and Ki372 were also allotted. They are, however, from the order Enterobacterales, family Enterobacteriacae, and genus *Kluyvera* [36]. The species *Citrobacter gillenii* and *C. freundii* (CG372, CG382, and CF402) also belong to the same family (Enterobacteriacae) [37]. The species *E. coli* EC361 also belongs to the same order and family, but to the genus *Escherichia* [38]. *Raoultella ornithinolytica* Ro381 belongs to the order Enterobacteriales, family Enterobacteriacae, and class Gammaproteobacteria; however, they belong to the phylum Pseudomonata [39]. *Hafnia alvei* is the species belonging to the phylum Pseudomonata, class Gammaproteobacteria, order Enterobacterales, family Hafniacae, and genus *Hafnia* [40]. The species *Leclercia* is a re-classified type of bacterium [41]. This bacterium was known as *Escherichia adecarboxylata*, and it is known as a human pathogen, which, however, is mostly susceptible to antibiotics [42]. Their taxonomy is also included in the phylum Proteobacteria, class Gammaproteobacteria, order Enterobacteriales, family Enterobacteriacae, and genus *Leclercia*. The same category is related to the species *Serratia fonticola* SF361. However, *Serratia* is allotted to the family Yersiniacae and the genus *Serratia* [43]. Pseudomonads belong to the same phylum and class, although they belong to the order Pseudomonales, family Pseudomonacae, and genus *Pseudomonas* [44]. In summary, five out of nine detected bacterial genera were from the phylum Proteobacteria (*Citrobacter, Pseudomonas*, *Serratia, Leclercia*, and *Escherichia*), and four genera belonged to the phylum Pseudomonata (*Kluyvera, Raoultella, Acinetobacter*, and *Hafnia*). However, all strains of detected species belong to the class Gammaproteobacteria. Seven genera (*Citrobacter, Escherichia, Kluyvera*, *Raoultella, Serratia, Hafnia*, and *Leclercia*) belong to the order Enterobacterales, and two genera (*Acinetobacter* and *Pseudomonas)* belong to the order Pseudomonales. The species of detected strains belong to five (5) families: Enterobacteriacae, with five genera (*Citrobacter, Escherichia, Kluyvera, Leclercia*, and *Raoultella*); Moraxellacae (*Acinetobacter*); Pseudomonacae (*Pseudomonas*); Yersiniacae (*Serratia*); and Hafniacae (*Hafnia*). In addition, detected bacteria belong to two phyla, Proteobacteria and Pseudomonata. *Kluyvera* spp. often occur in water and sewage and can be dangerous for immune-compromised patients [36,44,45]. However, for example, studying *Hafnia alvei* has been recommended in terms of two aspects: beneficial and non-requested. It can have importance because the production of a protein called caseinolytic protease B, which has been shown to be a mimetic of the hormone α-MSH, which is implicated in satiety [46]. From pathogenicity, *Hafnia* is considered a secondary pathogen via the nosocomial route. As has already been mentioned, *L. adecarboxylata* acts as a human pathogen; however, it is susceptible to antibiotics as effective therapy [46,47]. *Serratia* has been identified as an opportunistic pathogen. This means it can cause infection in individuals with weakened immunity or underlying medical conditions. This species is able to develop resistance to multiple antibiotics [48]. However, in our study, this strain, SF361, was susceptible to 9 out of 15 antibiotics. Johns et al. [38] reported a high prevalence of resistant *E. coli* in horses, and it is necessary to minimize their potential to be spread. Following this aspect, the use of postbiotics is promising, although in this study, only one strain *E. coli* was tested and it was postbiotic-resistant. However, in vivo, coliforms were reduced in animal experiments related to postbiotic–bacteriocin application [15]. Similarly, *Citrobacter* spp. is an opportunistic pathogen [48]. *Raoultella* spp. has been reported to be generally sensitive to treatment with carbapenems and β-lactamases [48]. *Pseudomonas* spp. is a bacterium widespread in various environments, and it has been shown that MALDI-TOF MS analysis is a reliable methodology for its identification, with up to 100% of isolates correctly identified [24]. Because there are more than 100 validated *Pseudomonas* species, there is also a problem with antibiotic resistance, and here, a novel approach seems to be postbiotics.

MALDI-TOF mass spectrometry has been indicated as a revolutionized procedure for bacterial clinical identification, as reported by Fedorko et al. [47]. In our study, broad variability in Gram-negative microbiota was detected. We did not include aeromonads; however, their species variability was presented in trout isolated from Slovakian water sources using MALDI-TOF MS identification in our previous study [12]. There were 9 different species determined among 25 isolated strains. Moreover, similarly to here, only two strains (aeromonads) were inhibited by the use of enterocins-postbiotics; the strains *Aeromonas bestiarum* R41/1 and R47/3 were inhibited with inhibitory activity of 100 AU/mL. Novotný et al. [8] described Gram-positive and Gram-negative pathogens from fishes, as well as Gram-negative species such as *Aeromonas* spp., *Campylobacter* spp., *Vibrio* spp., and *Salmonella* spp. They are also frequent agents that cause human infections. Regarding antibiotic susceptibility, the aeromonads formerly mentioned were similar to Gram-negative bacteria in terms of susceptibility [8]. However, there was noted resistance to ticarcillin of aeromonads, but in our study, strains were mostly susceptible to ticarcillin. In free-living trout, susceptibility to antibiotics should be obligatory, unlike farmed trout; farmed trout are commonly exposed to the use of antibiotics to prevent a loss caused by diseases [8,48]. Semwal et al. [9] reported the effectiveness of medicinal herbs against aeromonads in aquaculture. Phytotherapy-based techniques appear to be inexpensive and environmentally friendly, and bacteriocins (postbiotics) are. The plant extract from water hyacinth appeared to be effective against *Streptococcus iniae* in trout [9]. In general, there is an increasing interest in controlling antibiotic use. An alternative method to maintain a healthy microbial environment could be the use of postbiotics. However, more effectively, one could select postbiotics with a broad antimicrobial spectrum to eliminate Gram-negative bacteria as potential pathogens. Even *Hafnia alvei*, the strain HA4597, was reported to have an effect on cholesterol levels and induced a change in blood sugar levels, and it was indicated as a “precision probiotic” [49] with the potential to produce bacteriocin. Moreover, the antimicrobial effect of postbiotics has already been declared in vitro and in vivo [13,15]. Even the in vitro growth of fecal pseudomonads was inhibited using EntA/P, EntM, Ent55, and Ent2019 [50,51]. As reported, up to 9 out of 19 strains were inhibited with an inhibitory activity of 100 AU/mL. In vivo, the effect of postbiotic/enterocins was not only noted in broiler animals but also, e.g., in horses. After EntM administration for 21 days, an increased tendency of phagocytic activity in blood and a reduction in coliforms, campylobacters, and clostridiae was noted [15]. Selecting new bacteriocins produced by some of those species probably enables more effective inhibition of individual Gram-negative bacteria. Moreover, more effective inhibition could probably be achieved by using autochthonous strains that produce novel antimicrobial peptides, as declared, e.g., by Fan et al. [52]. Moreover, more intensive studies have been conducted to clarify the mode of action of postbiotics used in various applications [53]. For each case, our point of view represents an innovative approach.

## 5. Conclusions

Twenty-one (21) species strains isolated from the intestinal tract of trout in Slovakia were taxonomically allotted to 13 species and 9 genera, including the following species: *Acinetobacter calcoaceticus*, *Citrobacter gillenii*, *Citrobacter freundii*, *Escherichia coli*, *Hafnia alvei*, *Kluyvera cryocrescens*, *K. intermedia*, *Leclercia adecarboxylata*, *Raoultella ornithinolytica*, *Pseudomonas fragi*, *Ps. putida*, *Ps. lundensis*, *Ps. teatrolens*, and *Serratia fonticola*. The strains were mostly susceptible to antibiotics, and 14% of strains tested were susceptible to nine enterocins-postbiotics, with inhibitory activity up to 6400 AU/mL. An innovative approach to studying postbiotic use in aquaculture has been proposed. However, additional application experiments are required.

## Figures and Tables

**Table 1:** Gram-negative species originated from trout identified using MALDI-TOF mass spectrometry.

Species	Sample	Score	Indication
*A. calcoaceticus*	R36/2	2.225	AC362
*C. gillenii*	R37/2	2.319	CG372
*C. gillenii*	R38/2	2.330	CG382
*C. freundii*	R40/2	2.082	CF402
*E. coli*	R36/1	2.435	EC361
*H. alvei*	R33/1	2.108	Hal331
*H. alvei*	R37/3	2.126	Hal373
*H. alvei*	R38/2	2.273	Hal382
*H. alvei*	R40/1	2.487	Hal401
*H. alvei*	R28/1	2.051	Hal281
*K. intermedia*	R38/2	2.425	Ki382
*K. intermedia*	R37/1	2.326	Ki371
*L. adecarboxylata*	R38/1	1.788	LA381
*R. ornithinolytica*	R38/1	2.468	Ro381
*Ps. teatrolens*	R28/5	1.856	Pt285
*Ps. putida*	R39/1	2.300	Pp391
*Ps.fragi*	R28/4	1.887	Pf284
*Ps. fragi*	R30/2	1.947	Pf302
*Ps. lundensis*	R36/1	2.225	PL361
*S. fonticola*	R36/1	2.256	SF361

*A. calcoaceticus*, *Acinetobacter calcoaceticus*; *C. gillenii*, *Citrobacter gillenii*; *C. freundii*, *Citrobacter freundii*; *E. coli*, *Escherichia coli*; *H. alvei*, *Hafnia alvei*; *K. intermedia*, *Kluyvera intermedia*; *K. cryocresens*, *Kluyvera cryocrescens*; *L. adecarboxylata*, *Leclercia adecarboxylata*; *R. ornithinolythica*, *Raoultella ornithinolytica*; *Ps. teatrolens*, *Pseudomonas teatrolens*, *Ps. putida*, *Pseudomonas putida*; *Ps. fragi*, *Pseudomonas fragi*, *Ps. lundensis*, *Pseudomonas lundensis*, *S. fonticola*, and *Serratia fonticola*. Highly probable species identification (value score of 2.300–3.000), secure probable species identification/probable species identification (2.000–2.299), and highly probable genus identification (1.700–1.999).

**Table 2:** Antibiotic profile (phenotype) of Gram-negative trout-derived strains.

Strain	Carbenicillin (100 µg)	Erythromycin (15 µg)	Ampicillin (10 µg)	Trimetroprim (85 µg)	Ticarcillin (75 µg)	Clindamycin (2 µg)
AC362	R	R	R	R	+12	R
CG372	R	R	+10	R	+11	R
CG382	R	R	+12	R	+10	R
CF402	R	R	+10	R	+12	R
EC361	+15	R	+12	R	+15	R
Hal281	+14	+15	+21	R	+20	+19
Hal331	R	R	+11	R	R	R
Hal373	R	R	R	R	+12	R
Hal382	R	R	+10	R	+12	R
Hal401	R	R	R	R	+11	R
Kc383	R	R	+10	R	+10	R
Ki382	R	R	+10	R	+13	R
Ki371	+20	R	+20	+16	+26	R
LA381	R	R	R	R	R	R
Ro381	R	R	+11	R	+13	R
Pt285	+14	R	+15	+12	+19	R
Pp391	R	R	R	R	+11	R
Pf284	R	R	+11	R	+12	R
Pf302	R	R	+16	R	+11	R
PL361	+15	R	+17	+11	+20	R
SF361	R	R	R	R	+10	R

The strains were susceptible to mezlocillin (MEZ—75 µg), tetracycline (TE—30 µg), chloramphenicol (C—30 µg), piperacillin (100 µg), gentamicin (GN—120 µg), amikacin (AK—30 µg), azlocillin, mezlocillin (AZ, MEZ—75 µg), and azithromycin (AZM—15 µg). The strains were resistant to penicillin (P-10 IU); R—resistant; +—susceptible to inhibitory zone size in mm; AC36—*Acinetobacter calcoaceticus* AC362, CG—*Citrobacter gillenii* CG372, CG382, CF—*C. freundii* CF402; EC—*Escherichia coli* EC361; Hal—*Hafnia alvei*; Kc—*Kluyvera cryocrescens* Kc383, Ki—*Kluyvera intermedia* Ki382, Ki381; LA—*Leclercia adecarboxylata* LA381; Ro381—*Raoultella ornithinolytica*; Pt—*Pseudomonas teatrolens* Pt285; Pp391—*Ps. putida*, Pf 284, Pf302—*Ps. fragi*; PL361—*Ps. lundensis*, SF361—*Serratia fonticola*.

**Table 3:** *Enterocin postbiotics* used for treatment of Gram-negative strains and inhibition of 3 strains (arbitrary units per milliliter, AU/mL).

Strains	EntA/P	EntM	Ent4231	Ent55	Ent9296	Ent412	Ent2019	EntEM41	DurED26E/7
Hal281	6400	3200	200	3200	6400	6400	6400	3200	6400
Pp391	100	100	100	100	100	100	100	100	100
Pf284	100	100	100	100	100	100	100	100	100

Hal281, *Hafnia alvei* 281, Pp, *Pseudomonas putida* 391, Pf, *Ps. fragi* 284; EntA/P, enterocin-dipeptide produced by the environmental strain EK13-CCM7419 [27]; EntM, produced by the environmental strain *E. faecium* AL41-CCM8558 [18]; Ent412, produced by the horse-derived strain *E. faecium* EF 412 [28]; Ent4231, produced by the ruminal strain *E. faecium* CCM 4231 [29]; Ent55, produced by the poultry strain *E. faecium* EF55 [30]; Ent2019, produced by the rabbit-derived strain *E. faecium* 2019 -CCM 7420) [21]; Ent9296, produced by silage strain *E. faecium* EF9296 [31]; EntEM41, produced by ostrich strain *E. faecium* EM41 [22]; and Durancin ED26E/7, produced by the milk lump cheese-derived strain *E. durans* ED26E/7 [33]. They were prepared according to formerly reported protocols as referred. The initial activity of the precipitate was measured against the principal indicator *E. avium* EA5—our strain (Ent M, Ent 2019, Ent 9296—25,600 AU/mL, EntA/P, Ent 55—51,200 AU/mL, Ent 412—204,800 AU/mL, ED26E/7 Ent4231—6400 AU/mL, and EM41—800 AU/mL).

## Data Availability

Data will be provided by the corresponding author upon request.
